# The atherogenic index of plasma: A novel factor more closely related to non-alcoholic fatty liver disease than other lipid parameters in adults

**DOI:** 10.3389/fnut.2022.954219

**Published:** 2022-09-02

**Authors:** Jia Liu, Liyuan Zhou, Yu An, Ying Wang, Guang Wang

**Affiliations:** ^1^Department of Endocrinology, Beijing Chaoyang Hospital, Capital Medical University, Beijing, China; ^2^Medical Examination Center, Beijing Chaoyang Hospital, Capital Medical University, Beijing, China

**Keywords:** non-alcoholic fatty liver disease, atherogenic index of plasma, conventional lipids, discriminant model, adults

## Abstract

**Background and aims:**

The relationship of non-alcoholic fatty liver disease (NAFLD) with the atherogenic index of plasma (AIP) is unclear. This study aims to detect the association between AIP and NAFLD, compare the discriminative power of AIP with other lipid parameters for NAFLD, and establish a discriminant model using physical examination data.

**Methods:**

Participants aged over 20 years who underwent routine physical examination in Beijing Chaoyang Hospital from April 2016 to August 2020 were included. We categorized subjects based on hepatic ultrasound results and analyzed the association between NAFLD risk and AIP, conventional plasma lipids, remnant cholesterol (RC), triglyceride and glucose (TyG) index, and other atherogenic indices (*n* = 112,200) using logistic regression, restricted cubic spline regression, and receiver operating characteristic curve.

**Results:**

Out of the 112,200 subjects, 30.4% had NAFLD. The body weight index, plasma glucose, conventional lipids, TyG index, AIP, atherogenic coefficient (AC), and coronary risk index (CRI) were significantly higher, while HDL-C was lower (*p* < 0.001) in patients with NAFLD than those without NAFLD (all *p* < 0.001). Compared with conventional lipids, RC, TyG index, AC, and CRI, AIP had a stronger correlation with the risk of NAFLD (OR 6.71, 95% CI 6.23–7.22, *p* < 0.001) after adjusting confounders and presented a non-linear dose–response relationship (*p* < 0.0001). The optimal cut-off value of AIP was 0.05 and the area under the curve (AUC) was 0.82 (95% CI: 0.81–0.82) with high sensitivity and specificity. The AUC of the simplified three-variable NAFLD discriminant model was 0.90 in both the training set and the validation set.

**Conclusion:**

AIP was significantly associated with NAFLD and showed superior discriminative performance to other lipid parameters. These findings might help screen NAFLD in high-risk individuals and reduce the prevalence of NAFLD.

## Introduction

Non-alcoholic fatty liver disease (NAFLD) is the most common hepatic disease worldwide, with an estimated prevalence of 25.24% (1). In China, the prevalence of NAFLD has reached 29.81% and is still increasing (2). NAFLD is often paralleled with obesity, type 2 diabetes, hypertension, dyslipidemia, and metabolic syndrome (1). The complications of NAFLD include steatohepatitis, liver cirrhosis, and hepatocellular carcinoma, and thus increase the risks of all-cause mortality and liver-specific morbidity and mortality (3). Given the rapidly growing burden of NAFLD, it is challenging and necessary to identify non-invasive screening and diagnostic biomarkers to prevent the occurrence of NAFLD in high-risk populations (4).

NAFLD is characterized by excessive hepatic fat accumulation, which is induced by increased uptake of fatty acids (FA) and triglyceride (TG) from circulation, upregulated *de novo* lipogenesis, and the saturation of FA oxidation and very low-density lipoprotein (VLDL) secretion (5). However, evidence regarding the role of an individual lipid in promoting hepatic fat accumulation was controversial (6, 7). It has been reported that the proportion of atherogenic dyslipidemia including hypercholesterolemia, hypertriglyceridemia, and low level of high-density lipoprotein cholesterol (HDL-C) in patients with NAFLD ranges from 20 to 80% (8). Therefore, the conventional plasma lipids could not appropriately discriminate NAFLD. Cholesterol in VLDL, intermediate-density lipoprotein (IDL), and chylomicrons, also named remnant cholesterol (RC), was recently shown to be a factor related to NAFLD (9). Moreover, insulin resistance is another critical feature of NAFLD (10). The triglyceride and glucose (TyG) index was indicated as a surrogate index for insulin resistance (11). Emerging studies demonstrated that the TyG index was a marker for identifying NAFLD (12–15).

The atherogenic index of plasma (AIP) was a well-recognized predictive and prognostic biomarker for atherosclerotic cardiovascular disease (ASCVD) (16–18). In recent years, AIP was reported as a sensitive indicator of lipoprotein profiles to predict lipoprotein particle size (16). Tan et al. also showed that AIP was associated with insulin resistance (19). Therefore, AIP might play an important role in the development of NAFLD. Nevertheless, only a few previous studies investigated the relationship between NAFLD and categorical values of AIP in obese, non-obese, or small-sample populations and did not establish a discriminant model (20–22).

This study aims to explore the association of both categorical and continuous values of AIP with NAFLD and compare the discriminative power of AIP with other lipid parameters including conventional lipid profiles, RC, TyG index, atherogenic coefficient (AC), and coronary risk index (CRI) for NAFLD among a large-sample adult population in China. Simultaneously, we determined the optimal cut-off value of AIP to assist the screening and diagnosis of NAFLD by establishing a NAFLD discriminant model.

## Materials and methods

### Study design and populations

**FIGURE 1:**
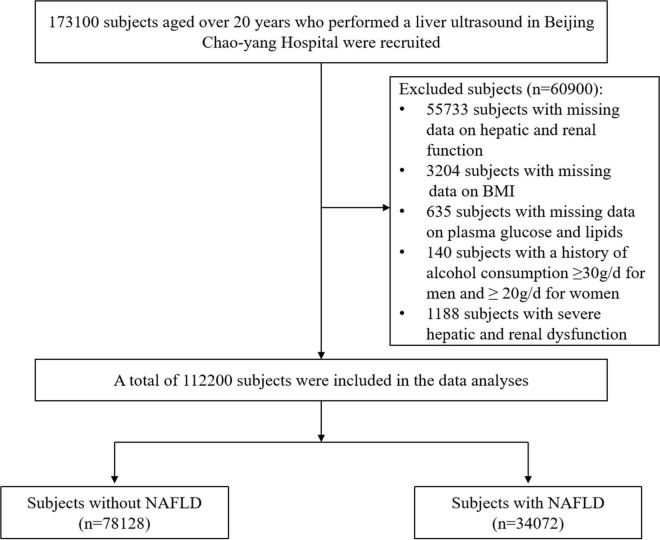
Flowchart of the cross-sectional study. BMI, body mass index; NAFLD, non-alcoholic fatty liver disease.

This cross-sectional study collected data from the routine health check-up (including hepatic ultrasound) of 173,100 subjects aged over 20 years in Beijing Chaoyang Hospital from April 2016 to August 2020 after excluding duplicate patients. Participants with missing data, a history of excessive alcohol consumption (≥30 g/d for men and ≥20 g/d for women), and severe hepatic and renal dysfunction were excluded. Finally, 112,200 subjects were included in the data analysis (Figure 1). We did not exclude the influence of hepatic disease history because less than 20% of the health examination in China included tests for viral hepatitis and autoimmune hepatitis (9).

This study was approved by the Ethics Committee of the Beijing Chaoyang Hospital affiliated with Capital Medical University and has been performed in accordance with the ethical standards laid down in the 1964 Declaration of Helsinki and its later amendments. Written informed consent was obtained from all participants.

### Diagnosis of non-alcoholic fatty liver disease

NAFLD was diagnosed by three well-trained and experienced clinicians *via* hepatic ultrasound and then the images were reviewed and confirmed by two experts in gastroenterology. The ultrasonic diagnosis for NAFLD included at least the first two of the following criteria: diffuse increases in liver echoes and liver–kidney contrast; liver brightness; vascular blurring; and deep attenuation (23).

### Measurements

Each participant underwent anthropometric and laboratory measurements. Body weight, height, and blood pressure were measured by trained nurses. Standing height and body weight were assessed by a wall-mounted stadiometer to the nearest 0.1 cm and 0.1 kg. Systolic blood pressure (SBP) and diastolic blood pressure (DBP) were the average of three consecutive measurements using a standard sphygmomanometer. Fasting plasma levels of glucose, total cholesterol (TC), triglyceride (TG), high-density lipoprotein cholesterol (HDL-C), low-density lipoprotein cholesterol (LDL-C), alanine aminotransferase (ALT), aspartate aminotransferase (AST), glutamyl transpeptidase (GGT), total protein (TP), albumin (ALB), total bilirubin (TBIL), creatinine (Cr), and uric acid (UA) were measured by an autoanalyzer.

### Calculations and definitions

Body mass index (BMI) was calculated by body weight in kilograms divided by height in square meters. Non–HDL-C was calculated by TC minus HDL-C, and RC was calculated as non–HDL-C minus LDL-C (24). TyG index, AIP, AC, and CRI were calculated according to the following equations: TyG = ln [fasting plasma glucose (mg/dL) *TG (mg/mL)/2]; AIP = log (TG/HDL-C); AC = non–HDL-C/HDL-C; CRI = LDL-C/HDL-C (16, 18, 25–27).

According to the World Health Organization (WHO) standard, overweight was defined as BMI ≥ 25 kg/m^2^ and obesity was defined as BMI ≥ 30 kg/m^2^. Based on the 2016 Chinese guideline for the management of dyslipidemia in adults (28), plasma lipids were categorized as follows: (1) TC: normal: < 5.2 mmol/L, marginal high: 5.2∼6.2 mmol/L, and high: ≥6.2 mmol/L; (2) TG: normal: <1.7 mmol/L, marginal high: 1.7∼2.3 mmol/L, and high: ≥2.3 mmol/L; (3) HDL-C: normal: ≥1.0 mmol/L, and low: <1.0 mmol/L; (4) LDL-C: normal < 2.6 mmol/L, marginal high: 3.4 ∼ 4.1 mmol/L, and high: ≥ 4.1 mmol/L; (5) non–HDL-C: normal < 4.1 mmol/L, marginal high: 4.1∼4.9 mmol/L, and high: ≥4.9 mmol/L. RC, TyG, AIP, AC, and CRI were divided into four quartile groups.

### Statistics

Categorical variables were defined as numbers (percentages) and were assessed by chi-square tests. The distribution of the continuous data was assessed by normal P–P plots. Normally distributed continuous data were expressed as mean ± standard deviation (SD). Non-normally distributed continuous variables were described as median and interquartile ranges and were analyzed after natural log-transformed. For continuous variables, comparisons between the NAFLD group and the non-NAFLD group were performed using Student’s *t*-tests.

When performing the association analysis of factors with NAFLD risk, the presence of NAFLD was defined as the dependent variable, and age, sex, BMI, SBP, DBP, ALT, AST, GGT, TP, TBIL, Cr, UA, glucose, TC, TG, HDL-C, LDL-C, non–HDL-C, RC, TyG index, AIP, AC, and CRI were used as independent variables for the univariate and multivariate logistic regression models. Restricted cubic spline regression (RCS) was used to assess the dose–response relationship between NAFLD risk and plasma lipids, RC, TyG, AIP, AC, and CRI after adjusting potential confounders. We used the receiver operating characteristic (ROC) curve to assess the screening and diagnostic performance of different factors and combined models, as well as the cut-off value of AIP for the prediction of NAFLD. The optimal cut-off value was determined according to the maximum Youden’s index. The two-sided *p* < 0.05 was considered statistically significant. SPSS version 22.0 (Chicago, IL, United States) and R version 4.0.2^1^ were used for all the statistical analyses.

## Results

### Characteristics of study participants

Among 112,200 participants aged over 20 years who performed hepatic ultrasound at the health check-up visit, 34072 (30.4%) had NAFLD. As shown in Table 1, patients with NAFLD presented significantly higher average or median age, BMI, SBP, DBP, ALT, AST, GGT, and TP. ALB, glucose, Cr, UA, TC, LDL-C, non–HDL-C, RC, TG, TyG index, AIP, AC, and CRI than those without NAFLD (all *p* < 0.001), while had lower levels of HDL-C (*p* < 0.001). NAFLD was more common in men than women.

**TABLE 1:** Comparison of clinical characteristics and metabolic parameters between subjects with NAFLD and those without NAFLD.

Characteristics	Non-NAFLD	NAFLD	*P*
N	78,128	34,072	N/A
Age (years)	40.7 ± 13.3	46.0 ± 13.0	<0.001
Sex, men, *n* (%)	33,302 (42.6%)	25,270 (74.2%)	<0.001
BMI (kg/m^2^)	22.9 ± 3.0	27.5 ± 3.3	<0.001
SBP (mmHg)	121.4 ± 16.5	132.3 ± 17.1	<0.001
DBP (mmHg)	72.3 ± 10.8	80.0 ± 11.7	<0.001
ALT (U/L)*	16.2 (12.4–22.6)	29.0 (21.0–42.5)	<0.001
AST (U/L)*	19.0 (16.9–22.9)	23.0 (19.6–28.9)	<0.001
GGT (U/L)*	15.0 (11.0–22.3)	30.0 (20.9–46.0)	<0.001
TP (g/L)	74.8 ± 4.0	75.4 ± 4.0	<0.001
ALB (g/L)	46.4 ± 2.6	46.9 ± 2.5	<0.001
TBIL (μmol/L)	14.9 ± 6.2	14.9 ± 6.0	0.123
Glucose (mmol/L)	5.21 ± 1.01	5.90 ± 1.70	<0.001
Cr (μmol/L)	64.7 ± 14.0	70.5 ± 14.1	<0.001
UA (μmol/L)	324.1 ± 84.7	403.6 ± 92.6	<0.001
TC (mmol/L)	4.75 ± 0.89	5.09 ± 0.95	<0.001
HDL-C (mmol/L)	1.43 ± 0.34	1.14 ± 0.25	<0.001
LDL-C (mmol/L)	2.62 ± 0.73	2.92 ± 0.81	<0.001
Non-HDL-C (mmol/L)	3.33 ± 0.86	3.94 ± 0.92	<0.001
RC (mmol/L)*	0.67 (0.46–0.89)	0.92 (0.63–1.25)	<0.001
TG (mmol/L)*	1.11 (0.82–1.53)	1.91 (1.40–2.65)	<0.001
TyG (mg/dL)^2^	8.45 ± 0.53	9.10 ± 0.60	<0.001
AIP	-0.09 ± 0.26	0.25 ± 0.28	<0.001
AC	2.33 (1.80–3.01)	3.49 (2.84–4.19)	<0.001
CRI	1.84 (1.41–2.37)	2.58 (2.09–3.11)	<0.001

Continuous data are expressed as mean ± SD or median with interquartile range and binary variable is expressed as n (%).

*Non-normally distributed continuous variables were compared after natural log-transformed. Data were analyzed by Student’s t-test. NAFLD, non-alcoholic fatty liver disease; BMI, body mass index; SBP, systolic blood pressure; DBP, diastolic blood pressure; ALT, alanine aminotransferase; AST, aspartate aminotransferase; GGT, glutamyl transpeptidase; TP, total protein; ALB, albumin; TBIL, total bilirubin; Cr, creatinine; UA, uric acid; TC, total cholesterol; HDL-C, high-density lipoprotein cholesterol; LDL-C, low-density lipoprotein cholesterol; RC, remnant cholesterol; TG, triglyceride; TyG, triglyceride glucose; AIP, atherogenic index of plasma; AC, atherogenic coefficient; CRI, coronary risk index.

### Associations of atherogenic index of plasma, conventional lipids, remnant cholesterol, triglyceride and glucose index, atherogenic coefficient, and coronary risk index with non-alcoholic fatty liver disease risk

In the univariate regression analysis, TC, TG, LDL-C, non–HDL-C, RC, TyG index, AIP, AC, and CRI were all significantly positively correlated with the risk of NAFLD, and HDL-C presented a negative correlation (Table 2). We further did multivariate logistic regression analyses to adjust potential confounding factors in two models and supported the above results (all *p* < 0.001), whereas the second and third quartiles of RC did not. By comparing the odds ratio (OR) value of the factors, we found that the positive effects of the highest quartile of AIP were the most obvious both before and after adjustment (unadjusted: OR 40.60, 95% CI 38.12–43.25; adjusted 1: OR 26.53, 95% CI 24.87–28.30; adjusted 2: OR 6.71, 95% CI 6.23–7.22; all *p* < 0.001), followed by the TyG index, AC, CRI, and TG. Similar patterns were identified in men and women, as well as lean subjects and subjects with overweight or obesity, when we did the multivariate regression analysis using the same adjusting factors except for sex or BMI (Table 3 and Supplementary Table 1), although the ORs were larger in women and lean subjects.

**TABLE 2:** Logistic regression analysis of lipid parameters with NAFLD (*n* = 112,200).

Variables	Unadjusted		Adjusted 1		Adjusted 2	
	OR (95%CI)	*p*	OR (95%CI)	*p*	OR (95%CI)	*p*
**TC (mmol/L)**						
<5.2	1.0		1.0		1.0	
≥5.2, <6.2	1.81 (1.76–1.87)	<0.001	1.65 (1.60–1.71)	<0.001	1.24 (1.19–1.29)	< 0.001
≥6.2	2.32 (2.21–2.42)	<0.001	2.15 (2.05–2.26)	<0.001	1.32 (1.24–1.41)	< 0.001
**TG (mmol/L)**						
<1.7	1.0		1.0		1.0	
≥1.7, <2.3	4.17 (4.12–4.42)	<0.001	3.41 (3.29–3.54)	<0.001	2.00 (1.92–2.09)	< 0.001
≥2.3	10.06 (9.70–10.44)	<0.001	7.51 (7.23–7.81)	<0.001	3.10 (2.96–3.25)	< 0.001
**HDL-C (mmol/L)**						
≥1.0	1.0		1.0		1.0	
<1.0	0.21 (0.20–0.22)	<0.001	0.28 (0.27–0.29)	<0.001	0.53 (0.50–0.55)	<0.001
**LDL-C (mmol/L)**						
<3.4	1.0		1.0		1.0	
≥3.4, <4.1	2.00 (1.93–2.07)	<0.001	1.75 (1.68–1.82)	<0.001	1.29 (1.23–1.36)	<0.001
≥4.1	2.70 (2.56–2.85)	<0.001	2.41 (2.28–2.56)	<0.001	1.52 (1.42–1.64)	<0.001
**Non-HDL-C (mmol/L)**						
<4.1	1.0		1.0		1.0	
≥4.1, <4.9	2.93 (2.84–3.03)	<0.001	2.41 (2.33–2.50)	<0.001	1.54 (1.48–1.61)	<0.001
≥4.9	4.10 (3.91–4.29)	<0.001	3.40 (3.23–3.57)	<0.001	1.70 (1.60–1.81)	<0.001
**RC (mmol/L)**						
Q1	1.0		1.0		1.0	
Q2	1.02 (0.98–1.07)	0.031	0.95 (0.91–1.00)	0.009	0.90 (0.85–0.95)	<0.001
Q3	1.67 (1.60–1.73)	<0.001	1.38 (1.32–1.44)	<0.001	1.04 (0.99–1.03)	0.163
Q4	4.50 (4.33–4.67)	<0.001	3.27 (3.14–3.40)	<0.001	1.63 (1.55–1.72)	<0.001
**TyG (mg/dL)^2^**						
Q1	1.0		1.0		1.0	
Q2	3.52 (3.32–3.74)	<0.001	2.93 (2.75–3.11)	<0.001	1.81 (0.69–1.94)	<0.001
Q3	9.57 (9.03–10.13)	<0.001	7.09 (6.69–7.52)	<0.001	3.06 (2.86–3.27)	<0.001
Q4	30.08 (28.41–31.85)	<0.001	20.07 (18.91–21.29)	<0.001	5.71 (5.33–6.13)	<0.001
**AIP**						
Q1	1.0		1.0		1.0	
Q2	4.15 (3.88–4.43)	<0.001	3.37 (3.15–3.60)	<0.001	1.99 (1.84–2.14)	<0.001
Q3	12.71 (11.93–13.53)	<0.001	9.09 (8.52–9.69)	<0.001	3.58 (3.34–3.85)	<0.001
Q4	40.60 (38.12–43.25)	<0.001	26.53 (24.87–28.30)	<0.001	6.71 (6.23–7.22)	<0.001
**AC**						
Q1	1.0		1.0		1.0	
Q2	3.52 (3.32–3.73)	<0.001	2.86 (2.69–3.03)	<0.001	1.83 (1.71–1.97)	<0.001
Q3	9.37 (8.86–9.01)	<0.001	6.61 (6.25–7.00)	<0.001	2.97 (2.77–3.17)	<0.001
Q4	25.77 (24.37–27.24)	<0.001	16.62 (15.69–17.60)	<0.001	4.82 (4.50–5.16)	<0.001
**CRI**						
Q1	1.0		1.0		1.0	
Q2	2.80 (2.66–2.94)	<0.001	2.29 (2.17–2.41)	<0.001	1.59 (1.50–1.70)	<0.001
Q3	6.21 (5.91–6.52)	<0.001	4.44 (4.23–4.67)	<0.001	2.37 (2.23–2.52)	<0.001
Q4	13.70 (13.05–14.37)	<0.001	8.95 (8.51–9.41)	<0.001	3.41 (3.21–3.63)	<0.001

Adjusted 1, for age and sex; adjusted 2, for age, sex, BMI, SBP, DBP, ALT, AST, GGT, TP, TBIL, Cr, UA, and glucose.

NAFLD, non-alcoholic fatty liver disease; OR, odds ratio; CI, confidence interval; TC, total cholesterol; HDL-C, high-density lipoprotein cholesterol; LDL-C, low-density lipoprotein cholesterol; RC, remnant cholesterol; TG, triglyceride; TyG, triglyceride glucose; AIP, atherogenic index of plasma; AC, atherogenic coefficient; CRI, coronary risk index.

**TABLE 3:** Multivariate logistic regression analysis of lipid parameters with NAFLD in men and women.

Variables	Men (*n* = 58,572)		Women (*n* = 53,628)	
	OR (95%CI)	*p*	OR (95%CI)	*p*
**TC (mmol/L)**				
<5.2	1.0		1.0	
≥5.2, <6.2	1.21 (1.15–1.27)	<0.001	1.26 (1.18–1.36)	<0.001
≥6.2	1.28 (1.19–1.39)	<0.001	1.30 (1.18–1.43)	<0.001
**TG (mmol/L)**				
<1.7	1.0		1.0	
≥1.7, <2.3	1.81 (1.72–1.91)	<0.001	2.35 (2.18–2.54)	<0.001
≥2.3	2.80 (2.65–2.96)	<0.001	3.80 (3.48–4.15)	<0.001
**HDL-C (mmol/L)**				
<1.0	1.0		1.0	
≥1.0	0.56 (0.53–0.59)	<0.001	0.39 (0.35–0.44)	<0.001
**LDL-C (mmol/L)**				
<3.4	1.0		1.0	
≥3.4, <4.1	1.18 (1.11–1.25)	<0.001	1.48 (1.36–1.60)	<0.001
≥4.1	1.36 (1.24–1.49)	<0.001	1.73 (1.53–1.94)	<0.001
**Non-HDL (mmol/L)**				
<4.1	1.0		1.0	
≥4.1, <4.9	1.45 (1.38–1.53)	<0.001	1.70 (1.57–1.83)	<0.001
≥4.9	1.64 (1.52–1.77)	<0.001	1.72 (1.55–1.90)	<0.001
**RC (mmol/L)**				
Q1	1.0		1.0	
Q2	0.92 (0.86–0.98)	0.011	0.84 (0.77–0.93)	<0.001
Q3	1.06 (1.00–1.14)	0.047	0.96 (0.88–1.05)	0.213
Q4	1.69 (1.59–1.80)	<0.001	1.45 (1.33–1.59)	<0.001
**TyG (mg/dL)^2^**				
Q1	1.0		1.0	
Q2	1.64 (1.50–1.79)	<0.001	1.97 (1.76–2.21)	<0.001
Q3	2.66 (2.44–2.89)	<0.001	3.48 (3.11–3.89)	<0.001
Q4	4.71 (4.31–5.14)	<0.001	7.38 (6.55–8.32)	<0.001
**AIP**				
Q1	1.0		1.0	
Q2	1.62 (1.46–1.79)	<0.001	2.26 (2.02–2.53)	<0.001
Q3	2.81 (2.55–3.10)	<0.001	4.19 (3.75–4.67)	<0.001
Q4	5.05 (4.59–5.57)	<0.001	8.97 (7.99–10.07)	<0.001
**AC**				
Q1	1.0		1.0	
Q2	1.46 (1.33–1.61)	<0.001	2.15 (1.94–2.38)	<0.001
Q3	2.21 (2.02–2.42)	<0.001	3.76 (3.40–4.17)	<0.001
Q4	3.66 (3.34–4.00)	<0.001	6.06 (5.44–6.75)	<0.001
**CRI**				
Q1	1.0		1.0	
Q2	1.31 (1.20–1.42)	<0.001	1.88 (1.70–2.08)	<0.001
Q3	1.80 (1.66–1.94)	<0.001	3.13 (2.84–3.45)	<0.001
Q4	2.54 (2.34–2.74)	<0.001	4.87 (4.40–5.39)	<0.001

Adjusted for age, BMI, SBP, DBP, ALT, AST, GGT, TP, TBIL, Cr, UA, and glucose.

NAFLD, non-alcoholic fatty liver disease; OR, odds ratio; CI, confidence interval; TC, total cholesterol; HDL-C, high-density lipoprotein cholesterol; LDL-C, low-density lipoprotein cholesterol; RC, remnant cholesterol; TG, triglyceride; TyG, triglyceride glucose; AIP, atherogenic index of plasma; AC, atherogenic coefficient; CRI, coronary risk index.

**FIGURE 2:**
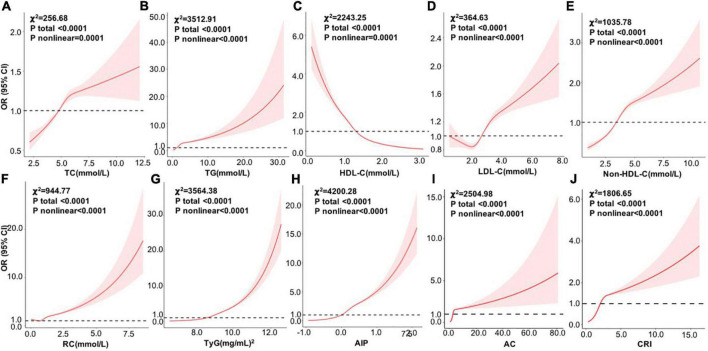
Dose–response relationships between variables and the risk of NAFLD by RCS analysis after adjusting age, sex, BMI, SBP, DBP, ALT, AST, GGT, TP, TBIL, Cr, UA, and glucose (*n* = 112200). **(A)** TC; **(B)** TG; **(C)** HDL-C; **(D)** LDL-C; **(E)** non-HDL-C; **(F)** RC; **(G)** TyG; **(H)** AIP, **(I)** AC; and **(J)** CRI. RCS, restricted cubic spline; OR, odds ratio; BMI, body mass index; SBP, systolic blood pressure; DBP, diastolic blood pressure; ALT, alanine aminotransferase; AST, aspartate aminotransferase; GGT, glutamyl transpeptidase; TP, total protein; TBIL, total bilirubin; Cr, creatinine; UA, uric acid; TC, total cholesterol; TG, triglyceride; HDL-C, high-density lipoprotein cholesterol; LDL-C, low-density lipoprotein cholesterol; RC, remnant cholesterol; TyG, triglyceride glucose; AIP, atherogenic index of plasma; AC, atherogenic coefficient; CRI, coronary risk index.

Figure 2 shows the dose–response relationships between the above factors and NAFLD risk by RCS analysis after adjusting age, sex, BMI, SBP, DBP, ALT, AST, GGT, TP, TBIL, Cr, UA, and glucose. Consistent with the above analysis by categorical values, a non-linear positive correlation was identified between NAFLD risk and TC, TG, LDL-C, non–HDL-C, RC, TyG index, AIP, AC, and CRI (all *p* total < 0.0001), among which AIP also showed the largest effects, followed by TyG, TG, and AC.

### Non-alcoholic fatty liver disease discriminant model

**FIGURE 3:**
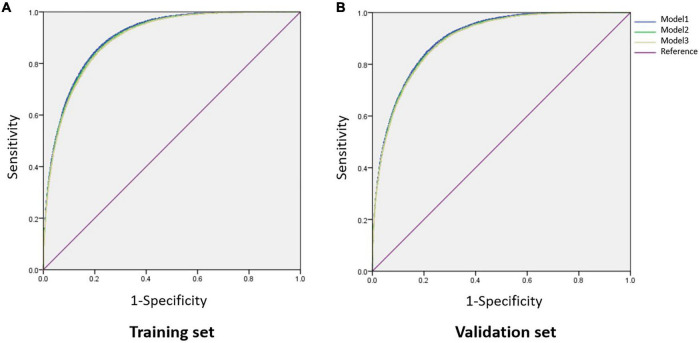
Comparison of AUCs among different combined models. **(A)** In the training set; **(B)** in the validation set. Model 1, BMI, ALT, GGT, TyG, AIP, UA, glucose, age, and DBP; Model 2, BMI, ALT, GGT, TyG, and AIP; Model 3, BMI, AIP, and ALT. AUC, area under the curve; BMI, body mass index; ALT, alanine aminotransferase; GGT, glutamyl transpeptidase; TyG, triglyceride glucose; AIP, atherogenic index of plasma; UA, uric acid; DBP, diastolic blood pressure.

To establish an optimal model to discriminate NAFLD, we divided the data into a training set and a validation set (7:3) and performed a ROC analysis on the individual factors which were related to NAFLD (Tables 1, 2) and the combined models. In the training set, the AUCs of BMI, AIP, AC, CRI, TyG, ALT, GGT, TG, HDL-C, and UA were larger than 0.70, while others were not. AIP had an AUC of 0.82 (95% CI: 0.81–0.82), slightly lower than 0.86 (95% CI: 0.85–0.86) of BMI, and the optimal cut-off value was 0.05 with the sensitivity of 0.78 and specificity of 0.70 (Table 4). In light of AIP and TyG presenting a stronger relationship with NAFLD than other lipid parameters, we only included these two lipid-related factors in the discrimination models together with other variables. Model 1 included TyG, AIP, BMI, ALT, GGT, UA, glucose, age, and DBP, and model 2 included TyG, AIP, BMI, ALT, and GGT. AUCs of model 1 and model 2 was 0.90 (95% CI: 0.90–0.91) and 0.90 (95% CI: 0.90–0.90), respectively. The simplified model 3 only included AIP, BMI, and ALT and had an AUC of 0.90 (95% CI: 0.90–0.90) with a sensitivity of 0.85 and a specificity of 0.78, among which AIP contributed the most (β = 3.01) (Table 4, Figure 3A, and Supplementary Table 2). Therefore, we preliminarily established a three-variable model for NAFLD discrimination.

**TABLE 4:** ROC analyses of metabolic parameters and combined models for NAFLD.

Parameters	Training set (*n* = 78,540)			Validation set (*n* = 33,660)		
	AUC (95%CI)	Sensitivity	Specificity	AUC (95%CI)	Sensitivity	Specificity
Model 1	0.90 (0.90–0.91)	0.87	0.77	0.90 (0.90–0.91)	0.85	0.79
Model 2	0.90 (0.90–0.90)	0.87	0.77	0.90 (0.89–0.90)	0.84	0.79
Model 3	0.90 (0.90–0.90)	0.85	0.78	0.90 (0.90–0.90)	0.84	0.79
BMI (kg/m^2^)	0.86 (0.85–0.86)	0.81	0.73	0.85 (0.84–0.85)	0.79	0.75
AIP	0.82 (0.81–0.82)	0.78	0.70	0.82 (0.82–0.83)	0.76	0.74
AC	0.79 (0.79–0.80)	0.77	0.68	0.79 (0.79–0.80)	0.74	0.71
CRI	0.75 (0.75–0.75)	0.75	0.63	0.76 (0.75–0.76)	0.74	0.64
TyG (mg/dL)^2^	0.80 (0.80–0.81)	0.76	0.70	0.81 (0.80–0.81)	0.80	0.66
ALT (U/L)	0.80 (0.80–0.80)	0.77	0.68	0.80 (0.80–0.81)	0.77	0.69
GGT (U/L)	0.80 (0.79–0.80)	0.77	0.70	0.80 (0.79–0.80)	0.78	0.68
TG (mmol/L)	0.79 (0.79–0.79)	0.74	0.70	0.80 (0.79–0.80)	0.76	0.69
HDL-C (mmol/L)	0.75 (0.75–0.76)	0.75	0.63	0.76 (0.75–0.76)	0.72	0.67
UA (umol/L)	0.74 (0.74–0.75)	0.75	0.61	0.75 (0.74–0.75)	0.76	0.61
Non-HDL-C (mmol/L)	0.70 (0.70–0.70)	0.72	0.58	0.69 (0.69–0.70)	0.72	0.57
AST (U/L)	0.70 (0.69–0.70)	0.62	0.67	0.70 (0.70–0.71)	0.62	0.68
DBP (mmHg)	0.69 (0.69–0.70)	0.67	0.62	0.68 (0.67–0.69)	0.68	0.59
SBP (mmHg)	0.69 (0.69–0.69)	0.66	0.62	0.69 (0.68–0.69)	0.65	0.62
Glucose (mmol/L)	0.68 (0.68–0.68)	0.59	0.68	0.66 (0.66–0.67)	0.59	0.66
RC (mmol/L)	0.67 (0.67–0.68)	0.60	0.68	0.67 (0.66–0.67)	0.59	0.67
Age (years)	0.63 (0.63–0.64)	0.67	0.53	0.62 (0.61–0.62)	0.67	0.49
Cr (umol/L)	0.63 (0.62–0.63)	0.69	0.53	0.62 (0.62–0.63)	0.67	0.55
LDL-C (mmol/L)	0.62 (0.61–0.62)	0.64	0.55	0.62 (0.62–0.63)	0.62	0.57
TC (mmol/L)	0.61 (0.61–0.62)	0.63	0.53	0.60 (0.59–0.61)	0.57	0.58
ALB (g/L)	0.56 (0.55–0.56)	0.58	0.50	0.56 (0.55–0.56)	0.57	0.51
TP (g/L)	0.55 (0.54–0.55)	0.57	0.50	0.55 (0.55–0.56)	0.55	0.53

Model 1, BMI, ALT, GGT, TyG, AIP, UA, glucose, age, and DBP; Model 2, BMI, ALT, GGT, TyG, and AIP; Model 3, BMI, AIP, and ALT.

ROC, receiver operating characteristic curve; AUC, area under the curve; CI, confidence interval; NAFLD, non-alcoholic fatty liver disease; BMI, body mass index; AIP, atherogenic index of plasma; AC, atherogenic coefficient; CRI, coronary risk index; TyG, triglyceride glucose; ALT, alanine aminotransferase; GGT, glutamyl transpeptidase; TG, triglyceride; HDL-C, high-density lipoprotein cholesterol; UA, uric acid; AST, aspartate aminotransferase; DBP, diastolic blood pressure; SBP, systolic blood pressure; RC, remnant cholesterol; Cr, creatinine; LDL-C, low-density lipoprotein cholesterol; TC, total cholesterol; TP, total protein; ALB, albumin.

We further validated the discriminant model in the validation set and showed that the three-variable model also had an AUC of 0.90 (95% CI: 0.90–0.90) with a sensitivity of 0.84 and a specificity of 0.79 (Table 4 and Figure 3B), which indicated that this model was stable. The optimal cut-off value of AIP was also 0.05 with an AUC of 0.82 (95% CI: 0.82–0.83) in this dataset (Table 4).

## Discussion

Using large-sample physical examination data, we uncovered that AIP was a superior NAFLD discriminator in the Chinese population than other lipid parameters, especially in women and lean subjects. Additionally, we determined the optimal cut-off value of AIP and used AIP as a new discriminator to establish a three-variable discriminant model (model 3) for NAFLD in both the training set and the validation set.

NAFLD has become the leading liver disease worldwide and is characterized by disorders of lipid metabolism and insulin resistance. AIP, a calculated lipid parameter, was shown to be a novel surrogate biomarker for insulin resistance (19). Nevertheless, the relationship between AIP and NAFLD was unclear and studies investigating the relevance of AIP with NAFLD were few and limited. Wang et al. first explored the relationship between AIP and NAFLD in 538 obese subjects. They divided the subjects into three groups based on AIP levels: low (<0.11), intermediate (0.11–0.21), and high (>0.21) groups. The results showed that the NAFLD risk increased by 4.37-folds in the high AIP group compared with the low AIP group after adjusting covariates. The AUC of AIP was 0.718 (95%CI 0.670–0.766) (20). Additionally, Dong et al. demonstrated a strong relationship between AIP and NAFLD in non-obese Chinese and Japanese subjects and showed the best cut-off value of AIP for NAFLD discrimination was 0.005 in the Chinese group and -0.220 in the Japanese group (22). Xie et al. evaluated the association between AIP and fatty liver among 7,838 adult participants and showed that the risk of fatty liver was increased in patients with the higher quartiles of AIP compared with those with the first quartile of AIP, particularly in women and youths. AIP showed a high ability for fatty liver prediction (AUC = 0.810) (21). Wen Jie also investigated the relationship between AIP and NAFLD in 160 patients with NAFLD and 160 healthy controls and showed that AIP was the risk factor for NAFLD (OR = 2.070, 95%CI = 1.785–2.732, *p* < 0.01). The AUC of AIP was 0.899 with a sensitivity of 0.829 and a specificity of 0.881 (29). Recently, Samimi et al. analyzed the association between AIP and metabolic-associated fatty liver disease (MAFLD) in patients with type 2 diabetes and found that the prevalence of MAFLD was significantly increased in patients with higher AIP quartiles (*p* < 0.001). They also showed a cut-off value of 0.54 for AIP in predicting MAFLD (sensitivity = 0.578, specificity = 0.544) (30). Among them, the population characteristics and the cut-off value, and AUC of AIP for NAFLD discrimination were inconsistent. Our study reached similar conclusions to these cross-sectional studies and showed a strong association of categorical AIP with NAFLD among 112,200 adult subjects, especially in women and lean patients. A non-linear positive association of continuous level of AIP with NAFLD was also observed. AIP presented a large discriminative performance (AUC = 0.82) for NAFLD in both the training dataset and the validation dataset. The optimal cut-off value of AIP was 0.05 with high sensitivity and specificity.

In addition to investigating the relationship between AIP and NAFLD risk, the present study first compared the discriminative power of AIP with the conventional plasma lipids, RC, the novel lipid-related indicators TyG index, and other atherogenic indices including AC and CRI. Similar to obesity and metabolic syndrome, NAFLD is also characterized by an atherogenic lipid profile including elevated TG and LDL-C levels and decreased HDL-C (31). However, not all patients with NAFLD had dyslipidemia (8). Consistently, previously published studies and our study showed a limited discriminative power of the conventional lipids for NAFLD (20, 21). In the past few years, remnant cholesterol in VLDL, IDL, and chylomicrons gained growing attention as an independent predictor of adverse cardiovascular events (24). Cross-sectional studies in Australia, Rome, Japan, and China consistently showed a strong positive correlation between RC and NAFLD occurrence (9, 32–34). Additionally, the TyG index has been revealed to be closely related to insulin resistance and was considered a surrogate indicator of insulin resistance (26, 35). Given the essential role of insulin resistance in the development of NAFLD, published studies have confirmed that the TyG index was an effective biomarker for identifying NAFLD (12, 14). Our study showed that AIP was the best discriminator for NAFLD than all other lipid parameters, including RC, TyG, AC, and CRI.

The potential mechanism linking AIP to NALFD remains elusive. AIP, calculated as log (TG/HDL-C), is a comprehensive index of plasma lipid and is always related to cardiovascular diseases (18). Other than exhibiting lipid profiles, AIP could predict the size of lipoprotein particles (36). Additionally, AIP was associated with the severity of insulin resistance (19). These might partially explain the superior discriminative power of AIP for NAFLD. Moreover, the heterogeneity and severity of NAFLD were reflected by the changes in transcriptome and related pathways (37). The roles of the anti-aging gene *Sirtuin 1* (*Sirt1*) in appetite regulation, cell senescence and apoptosis, nuclear–mitochondria interaction, adipose tissue–liver crosstalk, and neuron proliferation have been uncovered (38, 39). Inactivation of *Sirt1* by unhealthy diets was involved in the development and progression of NAFLD, diabetes, and metabolic syndrome, in which epigenetic modifications might play important roles (40–42). Nutritional interventions to activate *Sirt1* were related to the reverse of NAFLD (39). The inverse relationship between AIP and *Sirt1* was demonstrated by Rkhaya et al. in patients with metabolic syndrome (43). Zhang et al. further showed that upregulated expression of *Sirt1* was necessary for the improvement of lipid profile and AIP by quercetin (44). Therefore, the inactivation of *Sirt1* may also be linked to the optimal cut-off value of AIP for screening NAFLD. More studies are required to explore the role of *Sirt1* in linking AIP to NAFLD in future, which might help reduce the prevalence of NAFLD and initiate early treatment and improving the patient quality of life.

Liver biopsy is still the gold standard for the diagnosis of NAFLD. Therefore, it is of great importance to establish discriminant models using non-invasive and convenient indicators available by health check-ups for early screening of NAFLD in high-risk populations. In light of the powerful discriminant role of AIP, to overcome the unstable power of a single factor for discriminating NAFLD, this study established a combined model including AIP, BMI, and ALT. Our simplified three-variable model (model 3) is simple for clinical application and has a high discriminative performance (AUC = 0.90), which is higher than the newest NAFLD discriminant model including waist circumference, BMI, GGT, RC, ALT, UA, HDL-C, and sex (AUC = 0.89) by Zhang et al. (9), the prediction model of BMI, TG, ALT, GGT, HDL-C, total bilirubin, and direct bilirubin (AUC = 0.861) by Cai et al. (7), and the prediction model combined with BMI, TG, GGT, age, sex, race, type 2 diabetes, and smoking history (AUC = 0.83) by Rodriguez et al. (45). This three-variable NAFLD discriminant model has been verified in our validation set and was shown to be stable. More large-sample studies are required to further validate our model in future.

This study still has some limitations. First, we did not perform a quantitative evaluation for the severity of fatty liver. Second, we could not obtain confounder data including diet, physical activity, and medication usage, which might affect the associations of AIP and other factors with NAFLD. Finally, viral and autoimmune hepatitis were not detected and ruled out in this study and might have some influences.

In conclusion, this is the first study to explore the association of both categorical and continuous values of AIP with NAFLD compared to other lipid parameters and suggest a more closely positive correlation between AIP and NAFLD risk than other lipid parameters in adults based on a large-sample physical examination data. The simplified three-variable discriminant model including AIP, BMI, and ALT could be used as a simple, useful, stable, and effective tool for the screening and managing of NAFLD. These findings would help reduce the prevalence of NAFLD, initiate early treatment, and improve the patient quality of life.

## Data availability statement

The original contributions presented in this study are included in the article/Supplementary material, further inquiries can be directed to the corresponding author/s.

## Ethics statement

The studies involving human participants were reviewed and approved by the Ethics Committee of the Beijing Chaoyang Hospital affiliated with Capital Medical University. The patients/participants provided their written informed consent to participate in this study.

## Author contributions

JL and LZ analyzed the data and drafted the manuscript. YA integrated the data. YW was responsible for recruiting subjects, collecting data, and revising the manuscript. GW contributed to the study design and data interpretation and reviewed the manuscript. All authors read and approved the final version of this manuscript for publication.

## Footnotes

1
http://www.r-project.org
