# Supplementary material for: The atherogenic index of plasma: A novel factor more closely related to non-alcoholic fatty liver disease than other lipid parameters in adults
Source: Front Nutr. 2022 Sep 2;9:954219. doi: 10.3389/fnut.2022.954219 (PMC9478109; doi:10.3389/fnut.2022.954219)
Supplement: Supplementary file 1 [file Data_Sheet_1.pdf]

Supplementary Material

Table S1. Multivariate logistic regression analysis of lipid parameters with NAFLD in lean subjects and subjects with overweight or obesity.

**Variables**	**Lean (n=67196)**		**Overweight or obesity (n=45004)**	
	**OR (95%CI)**	***p***	**OR (95%CI)**	***p***
**TC (mmol/L)**				
<5.2	1.0		1.0	
≥5.2, <6.2	1.28(1.21-1.37)	<0.001	1.18(1.12-1.24)	<0.001
≥6.2	1.35(1.22-1.48)	<0.001	1.26(1.17-1.37)	<0.001
**TG (mmol/L)**				
<1.7	1.0		1.0	
≥1.7, <2.3	2.23(2.08-2.39)	<0.001	1.81(1.71-1.91)	<0.001
≥2.3	3.71(3.44-4.00)	<0.001	2.67(2.52-2.83)	<0.001
**HDL-C (mmol/L)**				
<1.0	1.0		1.0	
≥1.0	0.50(0.46-0.54)	<0.001	0.55(0.52-0.59)	<0.001
**LDL-C (mmol/L)**				
<3.4	1.0		1.0	
≥3.4, <4.1	1.34(1.24-1.44)	<0.001	1.23(1.16-1.30)	<0.001
≥4.1	1.60(1.43-1.79)	<0.001	1.42(1.29-1.56)	<0.001
**nonHDL (mmol/L)**				

<4.1	1.0		1.0	
≥4.1, <4.9	1.64(1.53-1.76)	<0.001	1.43(1.36-1.51)	<0.001
≥4.9	1.78(1.62-1.95)	<0.001	1.59(1.47-1.72)	<0.001
**RC (mmol/L)**				
Q1	1.0		1.0	
Q2	0.90(0.83-0.99)	0.028	0.89(0.83-0.95)	0.001
Q3	1.16(1.06-1.26)	0.001	0.94(0.88-1.00)	0.067
Q4	1.83(1.69-1.99)	<0.001	1.45(1.36-1.54)	<0.001
**TyG (mg/dL)^{2}**				
Q1	1.0		1.0	
Q2	1.96(1.75-2.20)	<0.001	1.62(1.48-1.77)	<0.001
Q3	3.59(3.21-4.01)	<0.001	2.53(2.32-2.75)	<0.001
Q4	7.34(6.54-8.24)	<0.001	4.42(4.04-4.84)	<0.001
**AIP**				
Q1	1.0		1.0	
Q2	1.96(1.75-2.20)	<0.001	1.83(1.65-2.02)	<0.001
Q3	3.87(3.47-4.32)	<0.001	3.02(2.74-3.32)	<0.001
Q4	7.73(6.90-8.65)	<0.001	5.40(4.90-5.96)	<0.001
AC				
Q1	1.0		1.0	
Q2	2.34(2.01-2.60)	<0.001	1.62(1.48-1.78)	<0.001

Q3	4.54(4.09-5.02)	<0.001	2.46(2.26-2.68)	<0.001
Q4	8.02(7.22-8.91)	<0.001	3.97(3.64-4.33)	<0.001
CRI				
Q1	1.0		1.0	
Q2	1.99(1.80-2.19)	<0.001	1.42(1.31-1.55)	<0.001
Q3	3.39(3.09-3.72)	<0.001	2.06(1.90-2.22)	<0.001
Q4	5.33(4.85-5.86)	<0.001	2.97(2.75-3.21)	<0.001

Adjusted for age, sex, SBP, DBP, ALT, AST, GGT, TP, TBIL, Cr, UA, and glucose.

NAFLD, non-alcoholic fatty liver disease; OR, odds ratio; CI, confidence interval; TC, total cholesterol; HDL-C, high-density lipoprotein cholesterol; LDL-C, low-density lipoprotein cholesterol; RC, remnant cholesterol; TG, triglyceride; TyG, triglyceride-glucose; AIP, atherogenic index of plasma; AC, atherogenic coefficient; CRI, coronary risk index.

Table S2. Multivariate analysis for the 3 combined models.

Characteristics	Model 1			Model 2			Model 3		
	**β**	**OR (95%CI)**	***p***	**β**	**OR (95%CI)**	***p***	**β**	**OR (95%CI)**	***p***
BMI (kg/m^2)	0.329	1.39(1.38-1.40)	<0.001	0.360	1.44(1.43-1.44)	<0.001	0.360	1.43(1.42-1.44)	<0.001
AIP	2.514	12.35(10.16-15.02)	<0.001	1.480	4.41(3.83-5.07)	<0.001	3.010	20.37(18.97-21.87)	<0.001
ALT (U/L)	0.029	1.03(1.03-1.03)	<0.001	0.029	1.03(1.03-1.03)	<0.001	0.031	1.03(1.03-1.03)	<0.001
GGT (U/L)	0.001	1.00(1.00-1.00)	0.005	0.002	1.00(1.00-1.00)	<0.001			
TyG (mg/dL)^2	0.011	1.01(0.91-1.12)	0.840	0.803	2.23(2.09-2.39)	<0.001			
Glucose (mmol/L)	0.127	1.14(1.11-1.16)	<0.001						
UA (umol/L)	0.003	1.00(1.00-1.00)	<0.001						
Age (years)	0.019	1.02(1.02-1.02)	<0.001						
DBP (mmHg)	0.008	1.01(1.01-1.01)	<0.001						

Model 1: BMI, ALT, GGT, TyG, AIP, UA, glucose, age, and DBP; Model 2: BMI, ALT, GGT, TyG, and AIP; Model 3: BMI, AIP, and ALT.

OR, odds ratio; CI, confidence interval; BMI, body mass index; AIP, atherogenic index of plasma; TyG, triglyceride-glucose; ALT, alanine aminotransferase; GGT, glutamyl transpeptidase; TG, triglyceride; HDL-C, high-density lipoprotein cholesterol; UA, uric acid; DBP, diastolic blood pressure.
